# Low Concentration Fe-Doped Alumina Catalysts Using Sol-Gel and Impregnation Methods: The Synthesis, Characterization and Catalytic Performance during the Combustion of Trichloroethylene

**DOI:** 10.3390/ma7032062

**Published:** 2014-03-12

**Authors:** Carolina Solis Maldonado, Javier Rivera De la Rosa, Carlos J. Lucio-Ortiz, Aracely Hernández-Ramírez, Felipe F. Castillón Barraza, Jaime S. Valente

**Affiliations:** 1 Universidad Autónoma de Nuevo León, UANL, Facultad de Ciencias Químicas, Ave. Universidad S/N, Cd. Universitaria, San Nicolás de los Garza, Nuevo León 64451, Mexico; E-Mails: carolina.solisml@uanl.edu.mx (C.S.M.); carlos.lucioor@uanl.edu.mx (C.J.L.-O.); aracely.hernandezrm@uanl.edu.mx (A.H.-R.); 2 Universidad Autónoma de Nuevo León, UANL, Centro de Innovación, Investigación y Desarrollo en Ingeniería y Tecnología (CIIDIT), Km 10 de la nueva carretera al Aeropuerto Internacional de Monterrey, PIIT Monterrey, Apodaca, Nuevo León 66600, Mexico; 3 Universidad Nacional Autónoma de México, UNAM, CNyN, Km. 107 Carretera Tijuana-Ensenada, Ensenada, Baja California 22800, Mexico; E-Mail: ffcb@cnyn.unam.mx; 4 Instituto Mexicano del Petróleo, Eje Central L. Cárdenas 152, AP 14-805, 07730 Mexico, D.F., Mexico; E-Mail: jsanchez@imp.mx

**Keywords:** alumina, impregnation, sol-gel, trichloroethylene

## Abstract

The role of iron in two modes of integration into alumina catalysts was studied at 0.39 wt% Fe and tested in trichloroethylene combustion. One modified alumina was synthesized using the sol-gel method with Fe added *in situ* during hydrolysis; another modification was performed using calcined alumina, prepared using the sol-gel method and impregnated with Fe. Several characterization techniques were used to study the level of Fe modification in the γ-Al_2_O_3_ phase formed and to correlate the catalytic properties during trichloroethylene (TCE) combustion. The introduction of Fe *in situ* during the sol-gel process influenced the crystallite size, and three iron species were generated, namely, magnetite, maghemite and hematite. The impregnated Fe-alumina formed hematite and maghemite, which were highly dispersed on the γ-Al_2_O_3_ surface. The X-ray photoelectron spectra (XPS), FT-IR and Mössbauer spectroscopy analyses revealed how Fe interacted with the γ-Al_2_O_3_ lattice in both catalysts. The impregnated Fe-catalyst showed the best catalytic performance compared to the catalyst that was Fe-doped *in situ* by the sol-gel method; both had better catalytic activity than pure alumina. This difference in activity was correlated with the accessibility of the reactants to the hematite iron species on the surface. The chlorine poisoning for all three catalysts was less than 1.8%.

## 1. Introduction

The catalytic activity of aluminas depends on their physicochemical properties, which can be controlled by the preparation method [1–6]. Different methods create different surface properties on aluminas, even though the final crystalline structure is the same. The sol-gel method provides an attractive, convenient route to manipulate the structural and textural properties and purity of a compound [7–10]. One of the main advantages of metal oxide materials obtained using the sol-gel method is that the properties can be altered by manipulating any of the processing steps during the formation of the precursors. This fact allows for the homogeneous mixing of transition metal cations at a molecular level and enhances the formation of polycrystalline particles with special properties. Product purity is among sol-gel’s advantages, because this method enables the straightforward study of every variation performed and its effect on the materials.

Doping refers to modifying the structural and physicochemical properties of a material, usually by adding small quantities of some metal to obtain improvements in the catalytic properties. Doping is generally performed with less than 2% by weight of the metallic agent [11]. The doping of an inorganic matrix like that of alumina can be performed by adding small amounts of a metal during synthesis. This *in situ* procedure allows the doping agent to be uniformly dispersed and closely associated with the inorganic matrix. The result is the ability to adjust the overall surface properties, crystallographic arrangements and texture among other properties [12,13]. The classic, commercial method to dope an oxide catalyst with metallic agents is the incipient wetness impregnation technique. Impregnation consists of introducing the oxide catalyst (generally as a powder) into a solution containing the metallic agent. The oxide is then recovered with the metallic agent adsorbed through chemical and physical interactions to the oxide catalyst for drying [12,14]. Both methods of doping, *in situ* and impregnation, create interactions with the oxide matrix and, depending on the method, the doping agent is available at the surface to some extent [6].

To compare both methods, achieving the same weight percentage of doping and observing the catalytic activity and selectivity are necessary. Previously, we reported alumina prepared using the sol-gel method and doped with iron at 5 wt% or without iron doping, and then the performance of both catalysts was compared in the combustion of the chlorinated volatile organic compound (VOC) trichloroethylene [15]. Catalytic combustion is a good alternative to eliminate VOC emissions (substances that damage the ozone layer), because catalytic combustion is considered to be a sustainable technology [16] and catalyzes the reaction to the complete oxidation products CO_2_ and H_2_O [17–19]. Recent reports have described the characterization of alumina doped with Fe (greater than 5%) and characterized by techniques, such as X-ray diffraction (XRD) and scanning electronic microscopy (SEM) [15,20,21]. This work reports for the first time the role of iron at very low concentration in alumina (0.39%), using different characterization techniques. The main purpose of this work is to study the role of iron over the physicochemical properties of alumina and its catalytic performance in trichloroethylene combustion and the influence of iron incorporation using the two methodologies.

## 2. Results and Discussion

### 2.1. Thermal Analysis

**Figure 1.**
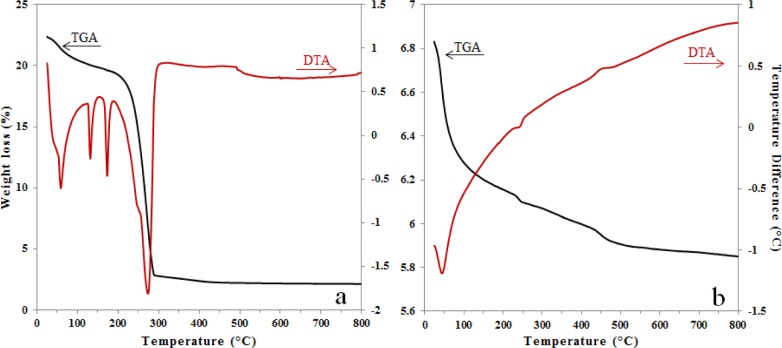
Thermogravimetric analysis (TGA) and differential thermal analysis (DTA) of (**a**) alumina doped with Fe *in situ* using the sol-gel method (AFSG) and (**b**) alumina doped with Fe by impregnation method (AFI).

The thermogravimetric analysis (TGA)/differential thermal analysis (DTA) results for the pure alumina xerogel were previously discussed and reported elsewhere [15]. Figure 1 shows the DTA and TGA in the same plot for xerogels of alumina doped with Fe *in situ* (Figure 1a) and alumina doped with Fe by impregnation (Figure 1b).

In Figure 1a, the first weight loss region (50–200 °C) is characterized by three endothermic events attributed to the physically adsorbed water (60 and 130 °C) and to formation of boehmite (173 °C). The second region (200–300 °C) has the greatest weight loss, due to the decomposition of nitrates, boehmite-alumina conversion and formation of iron oxide. The weight loss events were very similar to the pure alumina sample (not shown). The derivative of the DTA curve (not shown) allowed the identification of nitrates removal at 264 °C and boehmite conversion to γ-Al_2_O_3_ at 291 °C. A small shoulder at 246 °C was also visible in the derivative of the DTA and was identified as an endothermic event at 242 °C, corresponding to the dehydroxylation of Fe(OH)_3_ to γ-Fe_2_O_3_ (maghemite) [22]. Thus, the formation of iron oxide species allows the creation of the γ-Al_2_O_3_ phase at a lower temperature. Another short endothermic event at 495 °C without weight loss was observed on the TGA curve and attributed to the γ-Fe_2_O_3_ to α-Fe_2_O_3_ (hematite) phase transition [22]. Although the alumina phase of AFSG (alumina doped with Fe *in situ* using the sol-gel method) influenced the effects of strain release, the α-Fe_2_O_3_ particles were expected to be smaller than 60 nm.

In Figure 1b, the TGA shows an initial weight loss of 11% between room temperature and 230 °C, which can be attributed to physically absorbed water. Two pronounced slope changes were observed, the first from 230 to 260 °C (0.6%) and the second from 431 to 509 °C (1.3%). The first weight loss was related to the endothermic event at 242 °C; the decomposition of ferric acetate has been reported to occur between 240 and 300 °C and is accompanied by the desorption of acetone and carbon dioxide [23]. The second weight loss is related to an exothermic event at 445 °C; Kluchova *et al.* conducted detailed isothermal experiments at selected temperatures in the range of 320–400 °C for 1 h and proved that maghemite is the only formed Fe_2_O_3_ polymorph [24]; and Tae *et al.* reported that a weight loss occurring beyond 450 °C would be related to the decomposition of the residual organic acetate group [25,26]. The TGA/DTA of the AFI (alumina doped with Fe using the impregnation method) catalysts do not present other events (509–800 °C) and for the event at 445 °C this result could not be related with the hematite phase in accordance with what has been reported before.

### 2.2. X-Ray Diffraction (XRD) Patterns

**Figure 2.**
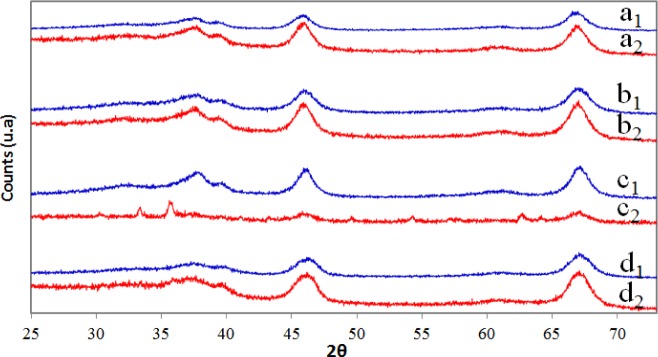
X-ray diffraction (XRD) patterns of the catalysts before (a_1_, b_1_, c_1_ and d_1_) and after combustion (a_2_, b_2_, c_2_ and d_2_), where the catalysts are identified as (**a**) A; (**b**) AFSG; (**c**) AFI; and (**d**) commercial sample of alumina (CA).

Figure 2 shows the XRD patterns obtained for the pure alumina (A), alumina doped with iron *in situ* using the sol-gel method (AFSG), alumina doped with iron by impregnation (AFI) and a commercial sample of alumina (CA) (commercial ketjen alumina) before and after trichloroethylene combustion. The γ-Al_2_O_3_ phase was the only crystalline structure identified (JCPDS 00-050-0741). The crystalline iron oxide species could not be detected due to the low content (<4 wt%) in AFSG and AFI. Table 1 presents the percentages of Fe determined in AFSG and AFI by X-ray fluorescence spectroscopy (XRFS). Table 1 also contains the mean crystallite size for all of the samples, calculated with the Scherrer equation [27].

The signal with the highest intensity was used (2θ ≈ 67) for all XRD patterns, which corresponded to the crystalline plane 440. The mean crystallite size value from the Scherrer equation for the AFSG sample was lower compared with pure alumina, while the value for AFI was higher. The inclusion of iron during the sol-gel process (AFSG) influenced the γ-Al_2_O_3_ mean crystallite size. The larger crystallite size for AFI compared to sample A could be explained by the calcination of sample A at 600 °C followed by impregnation with iron acetate solution and re-calcination at 600 °C, which led to sintering. The Fe or iron oxide species were highly dispersed in the γ-Al_2_O_3_ phase in the AFSG and could have spread into the crystal lattice to yield the crystallite size reported (Table 1). It is important to note that AFI was the only γ-Al_2_O_3_ catalyst affected after performing the trichloroethylene (TCE) oxidation.

### 2.3. Scanning Electronic Microscopy (SEM) Images and Energy Dispersive Spectra (EDS) Analysis

**Figure 3.**
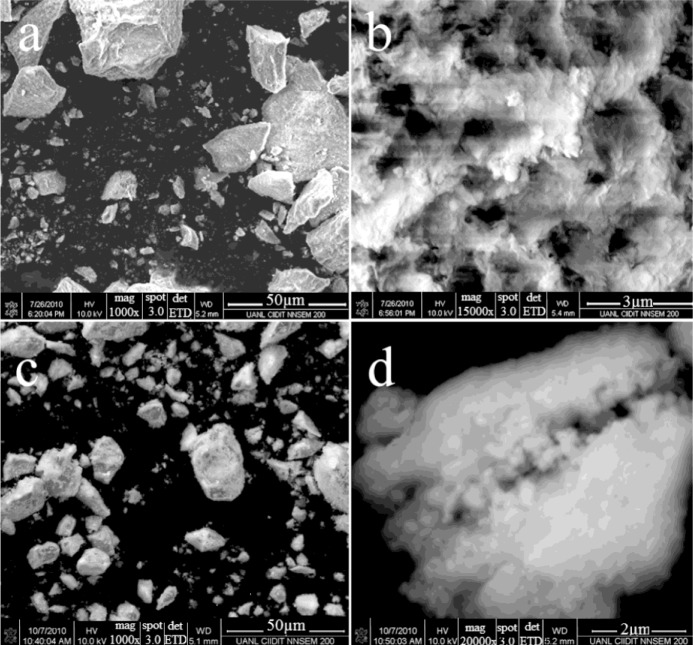
Scanning electronic microscopy (SEM) micrographs using two magnifications of AFSG (**a**,**b**) and AFI (**c**,**d**).

Figure 3 shows scanning electron micrographs of both modified alumina catalysts (AFSG and AFI) at two magnifications. For AFSG at 1000× (Figure 3a), a very regular morphology and a wide particle size distribution of grain was observed. The grains in the micrograph were formed from overlapping flakes; the low Fe content in AFSG led to this typical grain morphology for pure alumina. Figure 3b shows the same AFSG sample but at 15,000×, where the nanoparticles can be seen to have a very irregular size and shape. The individual particles were in the range of 200–600 nm. Some of these nanoparticles were agglomerated, and others were perfectly separated. In addition, a system of interconnected pores could be observed, which allowed the percolation of fluid and promoted permeability, favoring heterogeneous reactions. For AFI, the micrograph at 1000× (Figure 3c) showed grains of different sizes and a morphology similar to AFSG, but very small particles covering the surface of the grains were present. These particles were presumably impregnated iron oxide species. At 20,000× (Figure 3d), the AFI sample had nanoparticles that were less flat than the AFSG sample (Figure 3b). The nanoparticles were in the range of 200–610 nm. In particular, the nanoparticles were more connected with irregular borders than the AFSG nanoparticles, which could decrease the percolation of fluids compared to the AFSG sample.

Energy dispersive spectra (EDS) (data not shown) allowed the determination of the chemical composition of the samples [28,29]. These data were used to calculate the O/Al atomic ratios for all the fresh samples as shown in Table 1 [30]. The stoichiometric ratio of O/Al in the Al_2_O_3_ formula is 1.5; however, only the commercial catalyst had this value, while all of the synthetic fresh samples had higher values. The values of samples A, AFSG and AFI were 3%, 10.8% and 15.2%, respectively. This is higher than the stoichiometric value, likely due to excess oxygen from AlO(OH), iron oxides and surface nitrates. For example, γ-Al_2_O_3_ made by sol-gel synthesis was reported to be deficient (referring to atomic%) in aluminium [31]. In addition, γ-Al_2_O_3_ made by sol-gel synthesis was not totally dehydroxylated, with some hydroxyls from boehmite persisting in the alumina structure [15,31]. To preserve electron neutrality, some cationic defects must therefore be created in the structure. The number of cation defects must be equal to the number of OH ions left in the structure.

Table 1 also contains the O/Al ratios for all of the samples to be used in combustion tests with TCE (light-off curve tests). Importantly, EDS spectrometry allows for characterization of the first micrometres (≈ 2 μm) of the surface depth of solids [32]. The synthetic samples decreased the O/Al ratio by 20%, 29% and 23% for the A, AFSG and AFI samples, respectively. Notably, the fresh catalysts doped with Fe had a higher O/Al ratio on the surface (AFSG = 1.66 and AFI = 1.73) than without doping (A = 1.54 and AC = 1.5). The X-ray photoelectron spectra (XPS) spectral results of the O 1s show that relative area assigned to Al-O (Table 2) for the aluminas containing Fe are less (< 50%) than those without Fe. Therefore, the relative area for oxygen in Al-O was reduced due to the iron species decrease in the exposed alumina; however, the oxygen in iron oxide increases the presence of oxygen groups on the catalyst surface, resulting in a higher O/Al ratio, which favors TCE combustion. Iron oxide oxygen species have been reported to adsorb CO, transforming it into CO_2_ [33]. For this reason, the used catalysts, which had a higher activity (Fe-doped alumina), showed a decrease in the oxygenated groups in the EDS analysis, because these catalysts participated in the reaction forming CO_2_.

Furthermore, Table 1 reports the Cl wt% and degree of chlorine poisoning (AFSG was the most affected, followed by commercial alumina). The deposition of iron in the sol-gel sample (AFSG) proved to be more vulnerable to TCE combustion than the other samples, but this also meant a better interaction during the employment period (working time was 14 h for all of the samples). The attack of chlorine was very low, less than 1.8 wt% for all of the samples, which might have been due to the humid conditions favouring the Deacon reaction to form HCl.

The O/Al ratios for all of the samples were lower than the stoichiometric Al_2_O_3_ formula. The fresh samples were deficient in aluminium, and the used samples were deficient in oxygen, suggesting that TCE also reacted with oxygen in the solid samples. This oxygen might have come from the OH ions or reticular atoms in the γ-Al_2_O_3_ lattice in the case of the synthetic samples. The commercial sample had a slightly increased O/Al ratio, but contained chlorine atoms in the elemental analysis, indicating the formation of oxychlorine compounds.

### 2.4. High Resolution Transmission Microscopy (HRTEM)

**Figure 4.**
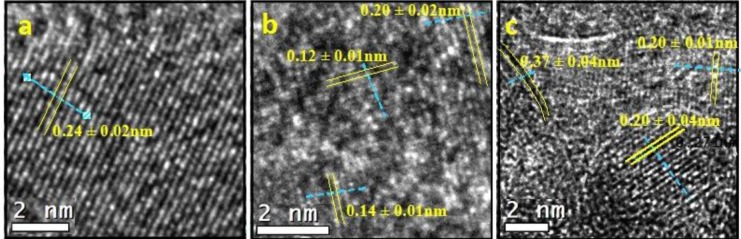
High resolution transmission microscopy (HRTEM) micrographs of (**a**) A; (**b**) AFSG and (**c**) AFI.

The images obtained by high resolution transmission microscopy (HRTEM) for the three synthetic samples are reported in Figure 4. The pure alumina sample (labelled as A) is included for comparison with the AFGS and AFI samples. Perpendicular lines were drawn for the interplanar distances (IDs) in the micrographs, and the average is reported on the micrographs.

For sample A (Figure 4a), a series of IDs revealed well-developed crystallite growth. This ID group occupied the central part of the image and measured up to 18 nm in length. One ID of 0.24 ± 0.02 nm and two of 0.25 ± 0.02 nm matched the (311) plane according to JCPDS 00-050-0741 for a γ-Al_2_O_3_ crystalline phase. Another zone with IDs of 0.15 ± 0.01 nm in the other direction was identified and matched the plane (511) of the same γ-Al_2_O_3_. At the top of the image in an area slightly out of focus, the background can be observed with accommodations for points in a hexagonal shape. This appearance was related to the carbonaceous layer on which particles from sample A were deposited. The pure alumina catalyst crystals were noted to grow well in a unidirectional mode.

Figure 4b shows the HRTEM for AFSG and different measured IDs. The four identified IDs were in different directions, so there were several directional growths of crystals. This growth yielded crystallite sizes smaller than 8 nm, which was consistent with the average AFSG crystallite size found in the XRD analysis (Table 1). The IDs measured were related to the crystalline phase of γ-Al_2_O_3_, but some of them might also correspond to crystalline phases of iron oxides. This result was the case for IDs of 0.20 ± 0.02 nm, which could be related to the (400) planes of iron oxides, such as magnetite or Fe_3_O_4_ (JCPDS 88-0866) and maghemite or γ-Fe_2_O_3_ (JCPDS 24-0081) (Fe_8_^III^ A[Fe_40/3_^III^ ⋄_7/3_]_B_O_32_, where ⋄ represents a vacancy, A is a tetrahedral positioning and B an octahedral positioning). The (202) plane of hematite or α-Fe_2_O_3_ (JCPDS 87-1165) could also be implicated, as could the (400) plane of γ-Al_2_O_3_ (JCPDS 00-050-0741). Furthermore, the IDs of 0.28 ± 0.05, 0.14 ± 0.01 and 0.21 ± 0.03 nm could correlate to different planes of the three iron oxides but also with some planes of γ-Al_2_O_3_. The IDs of 0.25 ± 0.01 and 0.12 ± 0.01 nm could only be related to the three types of iron oxide. At this point, the introduction of Fe *in situ* during synthesis by sol-gel to form the AFSG sample decreased the crystallite size compared to the pure alumina sample (A). The introduction of Fe also produced several directions of growth of the γ-Al_2_O_3_ crystallites along the borders. Some iron oxide phases may have grown epitaxially on some planes of the γ-Al_2_O_3_ phase. Another difference between sample A and AFSG was that the black spaces (vacancies) of A were very regular in size and frequency along the IDs. The Fe in the AFSG sample produced a variety of defects in the crystalline γ-Al_2_O_3_ phase.

Figure 4c shows the HRTEM of the AFI sample and contains a nanoparticle morphology identical to that found in previous work on 5 wt% Fe-doped Al_2_O_3_ by sol-gel for different iron oxides [15]. The morphology of the nanoparticles was roughly spherical, which was dissimilar to the morphology of pure alumina shown in Figure 4a. Thus, these roughly spherical particles could not correlate to the γ-Al_2_O_3_ phase. Different IDs were measured and identified. The IDs of 0.37 ± 0.03 nm and 0.27 ± 0.03 nm, found in an ellipsoidal particle (7.32 and 10.1 nm diameters). The ID of 0.37 ± 0.03 nm was related to the (012) plane for hematite and the (210) plane for maghemite. The ID of 0.27 ± 0.03 nm was related to the (104) plane for hematite and the (221) plane for maghemite. Any ID in the AFI HRTEM image could be correlated to a magnetite iron oxide phase. The iron oxide species found was hematite (α-Fe_2_O_3_) and maghemite is another phase that has been reported as a product of iron (II) acetate oxidation of γ-Al_2_O_3_. The latter matched the DTA analysis of the AFI sample. The IDs 0.20 ± 0.04 correlated with the plane (202) for the hematite and with the plane (400) for magnetite, maghemite and alumina.

**Figure 5.**
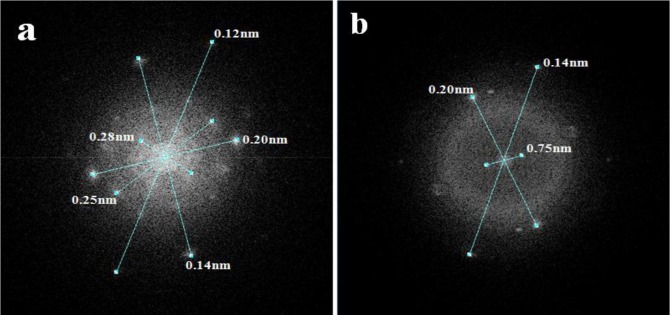
Fourier transform mode for (**a**) AFSG and (**b**) AFI.

Figure 5 presents the Fourier transform mode transmission electron microscopy (TEM) spot electrons for the AFGS and AFI samples. This analysis confirmed the crystalline nature of both catalysts and suggested some signals were related to different iron oxide phases. The incorporated iron in the AFSG sample could be present as iron oxide crystalline arrays, as substitutions or at interstitial positions in γ-Al_2_O_3_. The iron in the AFI surface was clearly presented as hematite and maghemite phases, but some Fe cations could have been introduced into γ-Al_2_O_3_ by diffusion from iron (II) acetate impregnation.

### 2.5. X-Ray Photoelectron Spectra (XPS) Measurements

**Figure 6.**
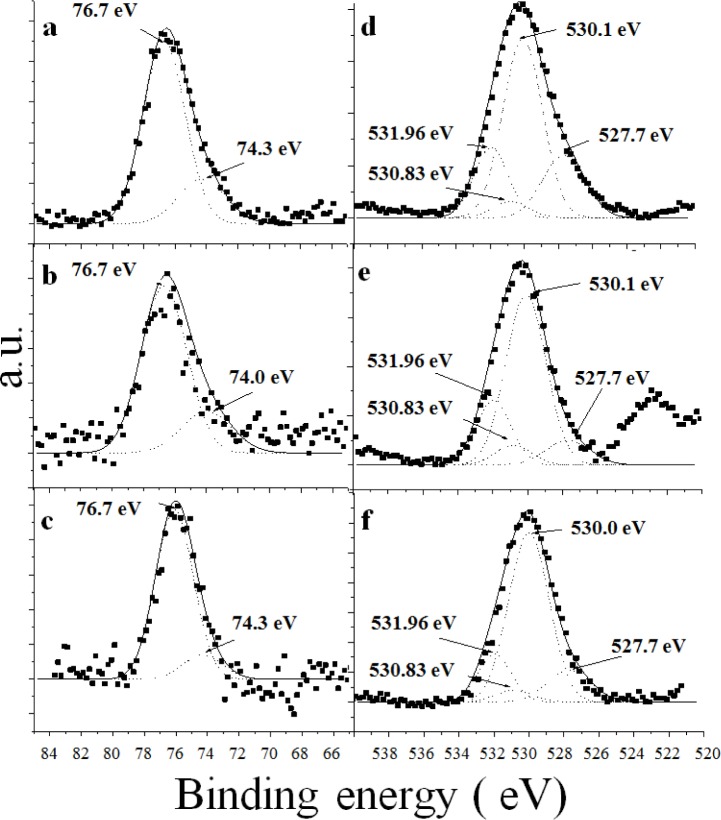
High-resolution Al 2p X-ray photoelectron spectra (XPS): (**a**) A; (**b**) AFSG; (**c**) AFI. O 1s XPS spectra; (**d**) A; (**e**) AFSG and (**f**) AFI.

Figure 6 presents the high resolution Al 2p XPS spectra for all of the synthetic catalysts. The catalysts A (Figure 6a) and AFSG (Figure 6b) had deconvoluted peaks at 76.7 eV and AFI at 76.1 eV (Figure 6c), which were attributed to aluminium cations in association with the surface-adsorbed nitrate when the sol-gel alumina samples were heated to high temperatures (600 °C) [34]. The AFI catalyst had the deconvolution with the lowest binding energy (BE) (Table 2, Al-*A*O_3_, where A is nitrogen of the NO_3_^−^). All the synthetic catalysts (A, AFSG and AFI) were made from a nitrate precursor to form the alumina phase at 600 °C, so a residual NO_3_^−^ anchored on the surface was not surprising. Importantly, to prepare the AFI catalyst, A was impregnated with iron acetate and calcined at 600 °C, which could explain the lower BE. The other deconvoluted Al 2p XPS peak (74.3 eV) for A and AFI was associated with an aluminium hydroxide species (marked as Al(OH)*_n_* in Table 2) [35]. For the AFSG sample, the deconvoluted XPS peak showed a BE below 74.0 eV. The *in situ* insertion of Fe species during the sol-gel synthesis of AFSG influenced the aluminium hydroxide species by substitution of Al^3+^ or insertion into interstitial sites in the lattice.

Table 2 shows the relative percentages of both types of deconvoluted Al 2p XPS peaks, Al-*A*O_3_ and Al(OH)*_n_*. The AFI sample had the lowest percentage of Al(OH)*_n_*, likely explained by either of the two following reasons: (a) the oxide iron species formed on the alumina surface blocked the Al(OH)*_n_* species or (b) the extra heat treatment evaporated the iron acetate impregnated over sample A and caused a greater degree of dehydroxylation.

Figure 6 presents the high-resolution O 1s XPS spectra for all of the synthetic catalysts. Four types of deconvoluted XPS peaks were resolved, and their BE values were assigned to the three samples. The first peak in the O 1s spectra at 531.96 eV was assigned to atomic oxygen in the γ-Al_2_O_3_ lattice [34,36]. The peak at 530.83 eV was assigned to the dissociatively adsorbed oxygen and implied a stronger bond between the adsorbed oxygen atoms and aluminium atoms [36]. The peak at 530.10 eV (for A and AFSG) was assigned to the hydroxyl oxygen (represented as O_2_-/hydroxyl in Table 2). This peak area was the largest of the four deconvoluted peaks. Only the peak in the AFI sample had a BE value below 530.0 eV [34]. The oxygen in the hydroxyl groups interacted with the iron oxide species over the alumina surface, as observed in the HRTEM and SEM images. The O-Al bond has been reported at 527.7 eV [37], which was deconvoluted in the O 1s XPS signal for the three catalysts. The relative percentage for the peak for AFSG and AFI decreased compared to A, indicating that the iron species decreased the amount of exposed alumina on the surface (Table 2).

Due to the low Fe concentration in the AFSG and AFI samples, the collected experimental XPS data were highly dispersed and the Fe 2p signal showed a very low intensity and signal-to-noise ratio; thus it was difficult to analyze its components by curve-fitting.

### 2.6. Fourier Transform Infrared (FT-IR) Spectroscopy Studies

**Figure 7.**
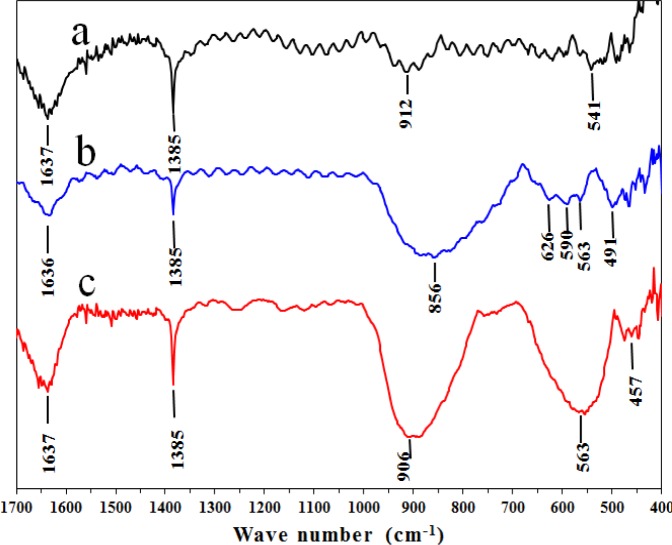
Fourier transform infrared (FT-IR) spectra for (**a**) A; (**b**) AFSG; and (**c**) AFI.

Figure 7 presents the Fourier transform infrared (FT-IR) spectra for the three synthesized catalysts in the low frequency range (400–1700 cm^−1^). A band at 1637 cm^−1^ was characteristic of the bending mode of H-O due to the deformation vibration mode of physisorbed water for the A and AFI samples. For AFSG, this band shifted to 1636 cm^−1^ as a consequence of the lower vibrational coupling energy of the H-O functional group. The sharp band at 1385 cm^−1^ in the three catalysts corresponded to nitrate ions and was consistent with the Al 2p XPS signal. The Al-O vibrations in the AlO_6_ octahedra and AlO_4_ tetrahedra were characterized by vibrational frequencies in the ranges of 500–700 and 700–900 cm^−1^, respectively. For the pure alumina catalyst (Figure 7a), the spectrum showed a band at 912 cm^−1^ associated with tetrahedral AlO_4_ and a band at 541 cm^−1^ corresponding to octahedral AlO_6_. Both bands showed the same intensity, which means equal amounts of both types of these sites in the γ-Al_2_O_3_ crystal.

The AFI catalyst (Figure 7c) displayed a broad, strong band at 906 cm^−1^ related to the tetrahedral AlO_4_ sites and a lower vibrational coupling energy than the A sample. This shift could have occurred upon heating of the AFI after iron impregnation. A broad band in the range of 1000–1200 cm^−1^ was reported for the Fe-O-Fe species of γ-Fe_2_O_3_ (maghemite) [38] and was observed in the TEM analysis. Hematite and maghemite nanoparticles were identified on the AFI catalyst surface, which might have contributed to the widening of the band reported at 906 cm^−1^ for this catalyst. Bands for maghemite were also identified at 795 cm^−1^ and 892 cm^−1^ [39]. The AFSG sample had a strong, broad band in the range of 700–900 cm^−1^, centered at 856 cm^−1^ and all three bands mentioned for maghemite. The band related to tetrahedral AlO_4_ for γ-Al_2_O_3_ had a lower wavenumber than usual for this type of site, suggesting the aluminium was in a tetrahedral site with a lower energy on the surface of the AFSG catalyst. For the AlO_6_ octahedral site in the AFI catalyst, a strong, broad band was observed at 563 cm^−1^. This site in the AFSG catalyst was one of three bands: 563, 590 or 626 cm^−1^. Clearly, the concentration of AlO_6_ octahedral sites in the AFI catalyst was greater than that for A or AFSG. At wavenumbers less than 640 cm^−1^, broad dual bands at 586 and 632 cm^−1^ were observed for the maghemite phase, and two strong, separated bands at 465 and 539 cm^−1^ were observed for the hematite phase [39]. The bands for both iron oxide materials were assigned in the AFSG spectra (Figure 7b) at 626, 590, 563 and 491 cm^−1^. For the AFI sample, the indicated band at 457 cm^−1^ was related to the hematite phase.

### 2.7. Textural Analysis from N_2_ Isotherms

**Figure 8.**
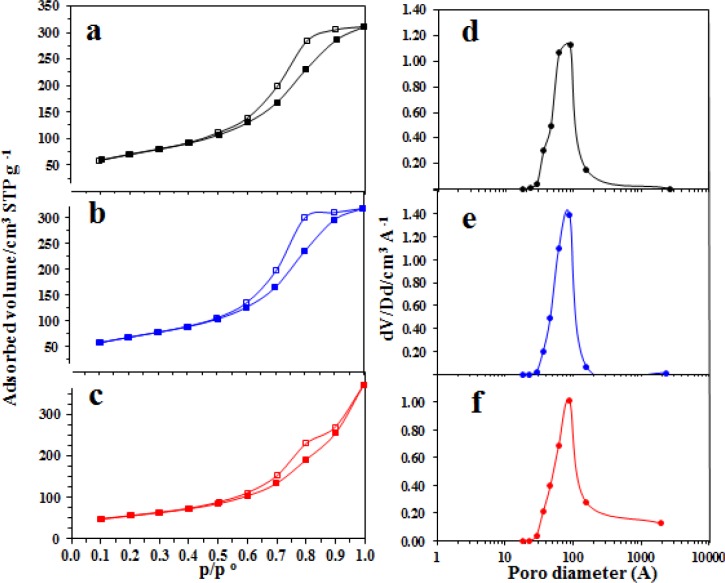
N_2_ adsorption/desorption isotherms at 77 K for (**a**) A; (**b**) AFSG and (**c**) AFI; pore size distribution (PSD) using the Barrett-Joyner-Halenda (BJH) model for the desorption isotherms for (**d**) A; (**e**) AFSG and (**f**) AFI.

Figure 8 shows the N_2_ adsorption/desorption isotherms at 77 K corresponding to each sample of the alumina substrates synthesized in this work. For the isotherms of N_2_, the filled symbols indicate adsorption, and the open symbols indicate desorption. The shapes of the adsorption isotherms for the three alumina samples, according to International Union of Pure and Applied Chemistry (IUPAC) classification, correspond to type IV isotherms [40–43]. These shapes were likely a result of the rapid decrease in the interaction potential energy with increasing distance from the molecules adsorbed on the surface.

The adsorption isotherms for typical A and AFSG samples (Figure 8a,b) were virtually identical, with only a very slight difference in the range of 0.9–1 p/p°. The adsorption isotherm for AFI also developed in a manner very similar to the other two samples, but in the range of 0.9–1 p/p°, the isotherm reached a higher adsorption volume and produced a greater quantity of macropores (>500 Å). Although iron oxide species were deposited on the surface of AFI, the pores smaller than 500 Å coalesced into macropores upon extra heat treatment at 600 °C.

Observing the adsorption and return effects together, the desorption isotherms showed hysteresis loops with intermediate behavior for types H1 and H2, reflecting the transition from H2 to H1 with increasing calcination temperature for the A and AFSG catalysts (Figure 8a,b) [44]. The most likely morphology is of large chambers delimited by narrow necks formed from pore cavities inside the solid mass by water vapour during the hydrothermal dehydration of the precursory gel. The AFI catalyst had a H3 type hysteresis loop, which is typically given for aggregates of plate-like particles or adsorbents containing slit pores. The hysteresis loop for AFI was also noticed to be more closed than for A or AFSG, likely a result of nearly ideal pores without interconnections. The desorption isotherms are the same for the three samples; the main difference is that the AFI catalyst has a different hysteresis loop, indicating the pores are blocked by impregnated Fe. In case of Fe incorporated *in situ*, this phenomenon was not observed, which indicates that fairly uniform cylindrical pores were obtained.

Figure 8 shows the pore size distribution (PSD) using the Barrett-Joyner-Halenda (BJH) model for the desorption isotherm for the three synthetic catalysts. The AFSG PSD was noticeably narrower compared to catalyst A. The integration of Fe in the sol-gel helped to form more homogenous microstructured material, which led to good catalytic performance in TCE combustion. Both catalysts A and AFSG were mesoporous material (20–500 Å porous diameter). The PSD for AFI had significant quantities of macropores (>500 Å), confirming the conclusions of the isotherm analysis.

Table 1 reports the average pore size calculated using the BJH desorption method and the Brunauer, Emmett and Teller (BET) method to calculate the specific area. As observed, the AFI catalyst had the highest average pore size and the lowest specificity among the catalysts. A lower performance for the AFI catalyst was expected for TCE combustion compared to A and AFSG, but the difference in iron oxide distribution might counteract this deficiency.

### 2.8. Mössbauer Analysis

**Figure 9.**
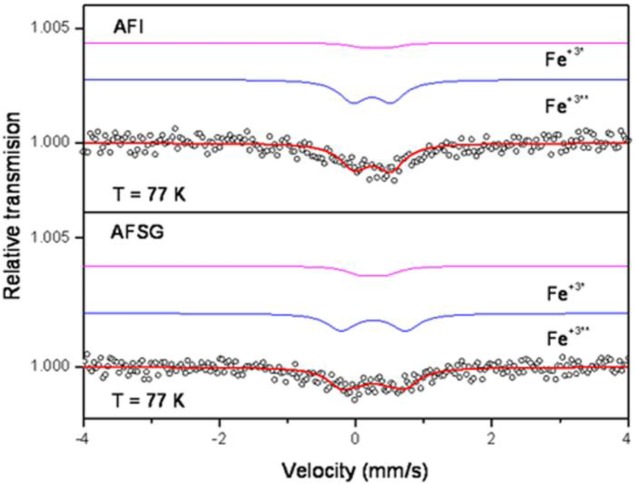
Mössbauer spectra at 77 K for (**a**) AFI and (**b**) AFSG.

Figure 9 shows the spectra for the AFSG 0.3% Fe and AFI 0.3% Fe catalysts, where the absorption was low because the Fe concentration was low. Both samples had two doublets and no magnetic components.

Table 3 shows the isomer shifts (IS) and the quadruple splittings (QS or QUA) for the alumina catalysts doped with Fe using sol-gel (AFSG) and impregnation (AFI). For the alumina doped with 0.3% Fe *in situ* using sol-gel (AFSG), the doublets had similar isomer shifts (*IS* = 0.25 mm/s), but different quadruple splitting values (*QS*_1_ = 0.94 mm/s and *QS*_2_ = 0.37 mm/s). The alumina doped with 0.3% Fe using impregnation (AFI) had different isomer shifts (*IS*_1_ = 0.23 mm/s and *IS*_2_ = 0.30) and quadruple splittings (*QS*_1_ = 0.56 mm/s and *QS*_2_ = 0.35 mm/s). The isomer shifts obtained for AFSG and AFI were characteristic of Fe^+3^ species (*IS* = 0.20 to 0.41 mm/s) [45,46] and could correspond to the hematite phase (α-Fe_2_O_3_) [47–49]. For the AFI sample, the QUA (*QS*_1_) and *IS* values were not fixed, and the fitting program iterated both until finding the optimal values, while for the AFS sample, the QUA (*QS*_2_) value was fixed as a constant. These results are the reason why the error for the AFSG samples is several hundred times greater than for the AFI sample. There were no magnetic properties (the sixth magnetic) because of the small crystal size. After adjustment, the two samples were found to have Fe^+3^ in different proportions. Specifically, one Fe^+3*^ assigned to the bulk and one Fe^+3**^ assigned to the surface. The AFSG catalyst contained 31% Fe^+3*^ and 69% Fe^+3**^, and the AFI catalyst contained 16% Fe^+3*^ and 84% Fe^+3**^. Moreover, the AFSG catalyst did not show changes in its IS, while the AFI catalyst increase from 0.23–0.30 mm/s could be associated with a decrease in the electron density around the iron nucleus; this change is related to a weakening of the Fe-O bonds [50]. AFSG had a QS (0.94 mm/s) greater than AFI (0.56 mm/s), suggesting that the Fe atoms in the AFSG catalyst were mainly on the alumina surface for AFSG and in the catalyst bulk or the nucleus particles for AFI [47,51]. The high QS for the AFSG sample also could correlate with some distortion in the support lattice due to Fe incorporation during the alumina synthesis.

### 2.9. Catalytic Tests

**Figure 10.**
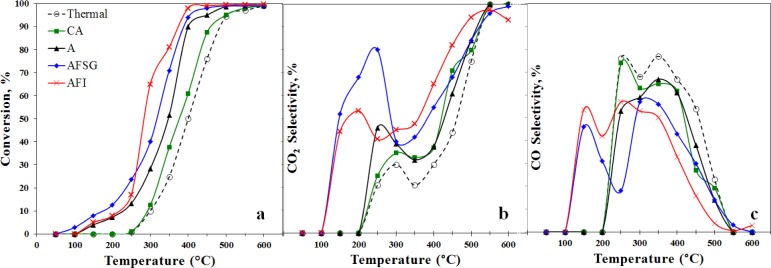
(**a**) Catalytic conversion, (**b**) selectivity toward CO_2_ and (**c**) CO during trichloroethylene (TCE) combustion; Thermal reaction (Thermal), CA, A, AFSG and AFI).

The catalytic tests were performed using the different catalysts synthesized in this work and a commercial alumina. Figure 10a shows the conversion as a function of temperature for TCE combustion (light-off curves). The conversion curves observed for the blank runs and homogeneous combustion are shown for comparison (thermal-labelled curve). At 150 °C, the three synthetic catalysts (A, AFSG and AFI) already converted TCE, while the commercial sample and the blank had not yet reacted.

Figure 10b and c shows the CO_2_ and CO selectivity, and only the iron-containing catalysts (AFSG and AFI) produced CO_2_ and CO at 150 °C. The AFSG and AFI catalysts did not adsorb TCE, but led to catalytic conversions at 150 °C. At 250 °C, all four catalysts produced CO_2_ and CO; however, AFSG had the best performance and demonstrated a CO_2_ selectivity of 80%. For comparison, the selectivities of the other catalysts were less than 47%. Therefore, the AFSG catalyst was superior up to 250 °C. Table 4 shows the percentage of conversion and selectivity (CO_2_ and CO) at temperatures from 150–400 °C, which is the range where the changes in the AFSG and AFI catalysts occurred.

The AFSG catalyst showed higher conversion than the AFI catalyst, below 250 °C. According to the characterization results, this could be attributed to the fact that AFSG included a narrower PSD, which promoted percolation and permeability, and a smaller mean crystallite size (calculated by the Scherrer equation), resulting in a greater number of meetings of the crystal planes in the grain boundary and thus more defects, creating physical sites for TCE and oxygen absorption. Another advantage of AFSG was the higher percentage of dissociated adsorbed oxygen over the surface, according to XPS characterization.

Identifying the isolated iron oxide nanoparticles from the TEM images (as observed for the HRTEM and SEM images for the AFI catalyst) on the surface of AFSG was not possible. However, the iron oxide planes were found to grow epitaxially on some planes of the γ-Al_2_O_3_ phase. Hence, the catalytic activity brought about by the introduction of iron to the AFSG catalyst was the result of a regular presentation of active sites over the catalyst surface.

The A and AFSG catalysts had almost the same upward slope, or typical steady-state extinction/ignition behavior that is common among strongly exothermic reaction systems [52]. Therefore, both catalysts likely have the same reaction mechanism, but the AFSG catalyst has a greater number of active sites provided by iron oxide species, as can be seen by FTIR spectra.

The AFI catalyst had the highest conversion at temperatures greater than 250 °C. Notably, from 250–350 °C, the slope changed twice. This behavior could be related to a change in the reaction mechanism over the surface of the AFI catalyst. However, the CO_2_ and CO production were nearly identical compared to the other catalysts (Figure 10b,c). This performance can be correlated with data obtained from the different characterization techniques used. The iron oxide nanoparticles identified over the surface of AFI by TEM, Mössbauer and FTIR, favors the oxygen mobility, but the oxygen species of iron oxide can adsorb CO, transforming it to CO_2_ [53]. The iron oxide nanoparticles seemed to contribute differently to the conversion of TCE compared to the other catalysts. Other authors have reported that iron oxide interaction with the support modify its chemical properties and catalytic performance on the volatile organic compounds oxidation [54,55].

This can be due to the iron showing a better dispersion over the AFI catalyst, according to Mössbauer analysis and also due to the higher concentration of iron oxide (hematite) and AlO_6_ octahedral sites, as showed in the FTIR spectra. Thus, the adsorption mechanism for oxygen may have changed between 250–350 °C, but the production of CO and CO_2_ was similar to the other catalysts.

The selectivity toward Cl_2_ and HCl was evaluated. At 500 °C, the catalysts with and without Fe showed a selectivity toward Cl_2_ of 0.5% and 1%, respectively. This can be correlated with the characterization results of EDS analysis, which showed that around 1.8 wt% Cl was deposited on the surface of the catalysts; thus we assumed that HCl was formed. The subproducts of incomplete combustion, such as tetrachloroethene (TTCE), 1,1,1-trichloroethane (TCA), 1,2-dichloroethane (DCA), suggest that some HCl reacts with TCE.

The formed products were similar to those reported in past works [56,57], but at very low concentrations. For example, the balance of chlorine at 300 °C for AFSG catalysts was: reacted TCE = 958.75 ppm, which gives 2876 ppm of chlorine. The products were: HCl = 2861 ppm, Cl_2_ = 0 ppm, TTCE = 2 ppm, TCA = 1 ppm, and DCA = 1 ppm, which gives a total of 2874 ppm of chlorine, a difference of 0.08%. Similar results were obtained for all the other catalysts.

The reaction rate (*r*_A_) was calculated considering an isothermal differential reactor model, as performed in a previous study [58]. At 300 °C, our alumina catalysts with iron had a higher reaction rate (Table 1) than the Fe-doped alumina (12.54 mg/g _Al2O3_) reported in a previous study, which showed a *r*_A_ of 1.06 × 10^−2^ mol_TCE_/(kg_Fe_-s) [15]. We calculated the reaction rate, but for comparison with the work of other authors, the light-off curve conversions are presented [56,57]. Evidently, the doping of the alumina catalyst with Fe led to a catalytic performance improvement.

However, the Fe incorporation using the impregnation method had a higher reaction rate. Oxygen atoms over the catalyst surface have been shown to play an important role in TCE combustion [59].

This finding can be related to the characterization results of Mössbauer spectroscopy. The Fe incorporated by impregnation(AFI) showed better dispersion than when Fe was incorporated *in situ* by sol-gel (AFSG), which could favor oxygen availability. Moreover, in XPS characterization results, we observed that the O 1s assigned to cations from O^−2^/hydroxides showed a lower binding energy for AFI (530 eV) than for A or AFSG (530.1 eV), because the oxygen in the hydroxyl groups interacted with the iron oxide species on the alumina surface. Furthermore, as already mentioned in previous sections, the iron oxide incorporated by both methods favors the O/Al ratio. Considering that the AFI catalyst has the highest oxygen content, the Fe_2_O_3_ is better dispersed in the bulk, and the link between Fe and O_2_ is weaker. The oxygen species are more available and dispersed in the catalyst, which favors a higher mobility of the species, mainly oxygen, and for this reason is more active [60].

The higher selectivity of CO_2_ than CO at low temperature (under 300 °C) for AFSG can be explained due to the role of hematite, which appears to proceed through two stages [61]: first, oxygen atoms adsorbed on the surface of hematite reacted with the gas phase CO according to an Eley-Rideal mechanism. Once that adsorbed oxygen was consumed, the surface oxygen from the lattice iron oxide was removed in a second stage involving CO adsorption and CO reactive desorption, thus generating surface oxygen vacancies. We previously reported the Langmuir-Hinshelwood (LH) mechanism of oxidation of TCE over zirconia doped with La and Fe [58], where molecular oxygen was considered to be dissociatively adsorbed onto an active site of the catalyst surface and then adsorbed oxygen atoms reacted with a nearby hydrocarbon on the same type of active site. The iron species on the alumina surface (AFI catalyst), and in particular the hematite phase, very possibly underwent an Eley-Rideal mechanism for the oxygen atoms adsorbed on the surface of hematite that reacted with TCE in the gas phase in addition to the LH mechanism for the alumina. After 350 °C, the catalysts all followed the same tendency of increased conversion. The AFI catalyst reached almost 100% TCE conversion at 400 °C, while the analogous thermal reaction (blank) did not reach a 100% conversion until 600 °C. Table 1 shows the T50% (temperature at which 50% conversion was attained) values for all four catalysts. AFI had the lowest temperature for 50% conversion. Using the reaction conditions of this work, the AFI catalyst had a lower T50% than in other recent studies using similar gas hourly space velocity (GHSV) values [58,60].

The significant differences of AFI with respect to the other synthetic catalysts is that AFI presented two iron oxide phases (maghemite and hematite), which were identified by TEM; but Mössbauer confirmed the hematite is distributed in the bulk of the catalyst and the Fe-O showed a weak bond, which favors the mobility of the oxygen. The AFI catalyst also showed an equilibrium in the AlO_6_ octahedric sites and AlO_4_ tetrahedric sites and a larger proportion of macropores in PSD (Figure 8). Figure 8 shows N_2_ adsorption/desorption isotherms at 77 K for (a) A, (b) AFSG and (c) AFI; PSD using the BJH model for the desorption isotherms for (d) A, (e) AFSG and (f) AFI.

## 3. Experimental Section

### 3.1. Catalyst Synthesis

Pure alumina (labelled A) was synthesized using the sol-gel method; the reagents used were aluminium nitrate nonahydrate (Al(NO_3_)_3_·9H_2_O 98%, Aldrich, St. Louis, MO, USA) as source of aluminium, ammonium hydroxide (NH_4_OH 5 mol·L^−1^, Aldrich) and deionized H_2_O. The volume and concentration of the reactants were as reported in our previous work [15]. For the alumina doped with Fe *in situ* using the sol-gel method (labelled as AFSG), 48.08 g of Al(NO_3_)_3_·9H_2_O and 48.08 g of NH_4_OH were dissolved in 600 mL of H_2_O during hydrolysis. Then, 0.080 g of iron(II) acetate (Fe(CO_2_CH_3_)_2_ 95%, Aldrich) was added to obtain 0.39 wt% Fe. Next, 400 mL of H_2_O and an excess of NH_4_OH were added to adjust the pH to 9. The solution was magnetically stirred for 24 h at room temperature. The gel obtained was dried at 80 °C to produce the xerogel, which was finally calcined at 600 °C. To prepare the alumina doped with Fe using the impregnation method (labelled as AFI), 2.5 g of pure alumina (catalyst A) was added to a solution of iron (II) acetate. The solution was prepared with 0.031 g of Fe(CO_2_CH_3_)_2_ dissolved in H_2_O to obtain a final concentration of 0.39 wt% Fe on the alumina. The mixture was dried at 80 °C, and finally the xerogel obtained was calcined at 600 °C. Commercial Ketjen alumina (CA) was used for comparison.

### 3.2. Characterization

Thermal analysis of the xerogels was performed using a TA Instruments SDT-2960 thermal analyzer a heating rate of 5 °C/min to 1000 °C in extra dry air flowing at 100 mL/min.

X-ray diffraction (XRD) was performed using a Siemens model D5000 series E04-0012 (Siemens Inc., Berlin, Germany), with Cu Kα radiation in the 2θ range of 5°–110° with a step of 0.020° every 4 s at 25 °C. The phases were identified using the Joint Committee on Powder Diffraction Standards (JCPDS) database.

N_2_ sorption isotherms at 77 K were recorded in an Autosorb-1 volumetric instrument (Quantachrome Co., Boynton Beach, FL, USA). Prior to adsorption runs, all of the samples were degassed overnight at 200 °C. Isotherms points (20 points): 0.1, 0.2, 0.3, 0.4, 0.5, 0.6, 0.7, 0.8, 0.9 and 0.995 (adsorption-desorption). The BJH method by desorption pore diameter (Barrett-Joyner-Halenda) and BET method by surface area (Brunauer, Emmet and Teller) were used. Ultra high purity (UHP) grade N_2_ and He gases were used.

Iron determination was performed by X-ray fluorescence spectroscopy (XRFS). The samples were heated to 950 °C. Then, the sample was analyzed in a sequential x-ray tube with a rhodium anode, using a RIGAKU 3270 spectrometer (Rigaku Industrial Corporation, Osaka, Japan).

SEM was performed using a FEI Nova Nano SEM 200 (FEI, Hillsboro, OR, USA) at 20 kV with a resolution of 1 μm; the EDS were collected with an EDAX Genesis XM4 detector (EDAX Inc, Mahwah, NJ, USA) for elemental analysis. All of the EDS spectra were corrected using ZAF correction, where *Z*, *A* and *F* are the matrix correction parameters, describing the atomic number effect (*Z*), absorption effect (*A*), and fluorescence effect (*F*).

For the high-resolution transmission electron microscopy (HRTEM), the sample was mixed with ethanol, and then was placed on the support films (lacey carbon type-A 300 mesh copper grids) and was dried. An FEI Titan G2 30-800 (FEI, Hillsboro, OR, USA) was used to obtain the micrographs.

X-ray photoelectron spectra (XPS) measurements were performed with Camac-3 equipment using one anode of Al as the excitation source with the following settings: λ = 1486.6 eV, non-monochromated, voltage acceleration = 15K eV and a filament current of 20 mA. The low-resolution spectra (survey) were in the range 1200–0 eV (binding energy) with a resolution of 3.0 eV. The high-resolution spectra of Al 2p and O 1s were evaluated with a resolution maximum of 0.8 eV, and the results were analyzed with the CAMAC-3 program. The C1s peak at 284.5 eV binding energy (BE) was used to compensate for the surface charge effects.

The FT-IR spectra for the samples, in the form of KBr mixed disks, were measured using a Nicolet™ 6700 spectrometer (Thermo Scientific, Waltham, MA, USA). The management software for sample analysis was OMNIC^TM^ (Thermo Scientific) in transmittance mode. The spectra were acquired with 32 scans using a resolution of 4 cm^−1^.

Mössbauer spectral analysis was performed with a conventional spectrometer (WissEL MRG-500, Wissenschaftliche Elektronik GmbH, Starnberg, Germany) at constant acceleration. The detection at 14.4 keV was performed with a proportional detector of krypton (Kr). The gamma radiation source was 25 m Ci of ^57^Co in a rhodium matrix. The spectrometer was operated in transmission mode. The analysis was performed at liquid nitrogen temperature (77 K = −196 °C) with 4 mm/s velocity. The isomer shift is given with respect to α-Fe. The spectra obtained were corrected using the Normos program.

### 3.3. Catalytic Tests

The catalytic tests were performed using a tubular continuous micro-reactor [15]. A flow of 100 mL/min of air with TCE at 1475 ± 25 ppm was passed through 1 g of catalyst into a tube with an inlet diameter of 0.6 cm. A gram of catalyst had 2 cm of packing volume, and the gas hourly space velocity (GHSV) was 10,610 h^−1^. The combustion temperature range was 50 to 600 °C with intervals of 50 °C. Chlorinated hydrocarbons (TCE, tetrachloroethylene, 1,1,1-trichloroethane and 1,2-dichloroethane were all supplied by Sigma-Aldrich as ACS reagent) with a known concentration were injected and determined using a gas chromatograph, model HP5890 II, equipped with an flame ionization detector (FID) and using a DB-624 capillary column (Agilent, Santa Clara, CA, USA) (ID 0.32 mm). The concentrations of CO_2_ and CO products were measured in line with an IR spectrometer (California Analytical, model 20, Orange, CA, USA). To measure the HCl and Cl_2_ concentrations, the effluent was bubbled into two containers with 150 mL of 0.0125 mol L^−1^ NaOH; the chlorine concentration was quantified using the colorimetric method of *N*,*N*-diethyl-p phenylenediamine (DPD) on a DR-890 instrument (HACH Company, Loveland, CO, USA) (8167 method). Chloride was measured using the Mohr Method [62]. To calculate the percentage of selectivity towards CO_2_ (φco_2_), the Equation (1) was used, where *C*co_2_, is the outlet concentration of CO_2_, and *TCE*_reacted_ is reacted TCE. This Equation (1) was also used for the selectivity towards CO by replacing the CO_2_ concentration. The selectivity to the different chlorinated products (φCl_2_ or φHCl) was calculated according to Equation (2), where *n* is the number of chloride atoms in the chloride product (Cl_2_ or HCl) and *C*_Clesp_ is the concentration of chlorine species:

$$\varphi {\text{co}}_{\text{2}}=100\frac{C_{{\text{co}}_{2}}}{2TCE_{\text{reacted}}}$$ (1)

$${\varphi \text{Cl}}_{\text{2}}\;or\;\varphi \text{HCL}=\frac{100nc_{\text{Clesp}}}{{3TCE}_{\text{reacted}}}$$ (2)

## 4. Conclusions

The role of iron in two different modes of integration was studied by incorporating Fe at a low concentration *in situ* during the sol-gel process or by impregnation of iron acetate solution into a pure alumina. For the AFSG sample, the inclusion of iron *in situ* by sol-gel retarded γ-Al_2_O_3_ crystallite growth and decreased crystallite size; the identified iron oxide phases were mainly maghemite and hematite. For the AFI catalyst, the growth was in a segregation form, and nanoparticles of iron oxide were deposited on the surface, which were identified as hematite and maghemite. The Fe species were introduced into γ-Al_2_O_3_ by diffusion, and this process favored their location on the bulk. Both Fe-doped aluminas had a better catalytic performance compared to non-doped conditions. AFSG was the best catalyst, showing a superior catalytic performance at temperatures less than 250 °C; above this temperature, the AFI catalyst was better. The nanoparticles of iron oxide, such as hematite, were located over the surface of γ-Al_2_O_3_ and modified the mechanism for temperatures above 250 °C to develop a better adsorption of oxygen molecules or atoms to promote CO_2_ selectivity. However, while the AFI catalyst exhibited a better catalytic performance above 250 °C, structural instability was present. Notably, chlorine poisoning using any of the three synthetic catalysts was less than 1.8%. Therefore, an improvement in resisting chlorine poisoning using all synthetic catalysts was achieved with AFSG being the most competitive during trichloroethylene combustion.

## Figures and Tables

**Table 1.** Characteristics of the alumina catalysts.

Sample	Crystallite size (nm) Fresh sample	Crystallite size (nm) Used sample	Fe (wt%)	*A*_BET_ ^a^ (m^2^/g)	Average pore size ^b^ (nm )	O/Al ^d^ Fresh sample	O/Al ^d^ Used sample	Cl ^d^ wt% Used sample	*T*_50%_ (°C)	mg_Fe_/g_Al2O3_	(*r*_A_) mol_TCE_/(kg_Fe_-s)
A	9	10	–	248	6.1	1.54	1.24	0.72	349	–	–
AFSG	7	11	0.37	242	6.1	1.66	1.18	1.78	315	3.7	1.18 × 10^−2^
AFI	11	16	0.39	195	8.8	1.72	1.32	1.01	285	3.9	1.82 × 10^−2^
CA	9	9	–	191	6.1	1.50	1.51	1.28	378	–	–

Note:

a BET surface area;

b BJH Method for desorption pore diameter;

c *T*_50%_ Temperature at 50% conversion of TCE;

d EDAX TSL^®^ advanced microanalysis solutions AMETEK. A: Pure alumina by the sol-gel method; AFSG: Alumina doped with Fe *in situ* by the sol-gel method; AFI: Alumina doped with Fe by impregnation; AC: Commercial Ketjen alumina.

**Table 2.** Deconvolution parameters for the alumina catalysts; FWHM: Full width at half maximum.

Sample	Cation peak. assignation		Position (eV)	FWHM (eV)	Relative area (%)
A	Al 2p	Al-*A*O_3_	76.70	2.7	79
		Al(OH)*_n_*	74.30	3.0	21

	O 1s	O/γ-Al_2_O_3_	531.96	2.0	19
		O_ads_	530.83	2.1	5
		O^2−^/hydroxyl	530.10	2.3	56
		O-Al	527.70	2.5	20

AFSG	Al 2p	Al-*A*O_3_	76.70	2.9	78
		Al(OH)*_n_*	74.00	3.2	22

	O 1*s*	O/γ-Al_2_O_3_	531.96	2.0	20
		O_ads_	530.83	2.1	7
		O^2−^/hydroxyl	530.10	2.3	63
		O-Al	527.70	2.5	10

AFI	Al 2p	Al-*A*O_3_	76.10	2.4	87
		Al(OH)*_n_*	74.00	2.5	13

	O 1s	O/γ-Al_2_O_3_	531.96	1.9	16
		O_ads_	530.83	2.1	4
		O^2−^/hydroxyl	530.00	2.3	67
		O-Al	527.70	2.5	13

**Table 3.** Mössbauer parameters of AFSG and AFI.

Parameters	AFSG	Error	AFI	Error	Unit
ISO (*IS*_1_)	0.25	±3.60E-02	0.23	±1.34E-04	mm/s
ISO (*IS*_2_)	0.25	±8.43E-02	0.30	±1.28E-04	mm/s
QUA (*QS*_1_)	0.94	±7.88E-02	♦	–	mm/s
QUA (*QS*_2_)	♦	–	0.35	±1.72E-04	mm/s
Fe^+3^*	31	–	16	–	%
Fe^+3^**	69	–	84	–	%

*IS*: isomer shifts; *QS*: quadruple splittings;

♦ *QS*_2_ value remained fixed at 0.374 mm/s for AFSG, while the *QS*_1_ was a value fixed of 0.56 mm/s for AFI, leading to a low χ^2^; Error = ±Error (1 × STD);

* doublets short = assigned to the bulk;

** doublets long = assigned to the surface.

**Table 4.** Trichloroethylene (TCE) conversion and CO_2_/CO selectivity.

Temperature (°C)	Conversion (%)					CO_2_, Selectivity (%)					CO, Selectivity (%)				

	Thermal	CA	A	AFSG	AFI	Thermal	CA	A	AFSG	AFI	Thermal	CA	A	AFSG	AFI
150	0	0	4	8	5	0	0	0	52	44	0	0	0	46	54
200	0	0	7	13	8	0	0	0	68	53	0	0	0	31	42
250	1	1	13	24	17	21	25	46	80	41	76	74	53	18	57
300	10	13	28	40	65	30	35	39	40	45	68	63	59	57	53
350	25	38	52	71	81	21	33	32	42	48	77	65	67	56	50
400	50	61	90	94	98	30	37	38	55	65	67	62	61	43	33
